# Optimizing thoracodorsal artery perforator flap outcomes in oncoplastic breast surgery: multidimensional assistive techniques mitigate learning curve and enhance feasibility

**DOI:** 10.1038/s41598-025-95073-z

**Published:** 2025-03-29

**Authors:** Zhipeng Hong, Zhihao Li, Xinhai Zhang, Chengye Hong, Liangqiang Li, Debo Chen

**Affiliations:** 1 Department of Breast Surgery, Affiliated Quanzhou First Hospital of Fujian Medical University, Quanzhou, 362000 Fujian Province P. R. China; 2 The School of Clinical Medicine, Fujian Medical University, Fuzhou, China; 3 Fudan University Shanghai Cancer Center Xiamen Hospital, Xiamen, 361000 P.R. China

**Keywords:** Thoracodorsal artery perforator flap, ICG, Oncoplastic breast surgery, BREAST-Q, Complications, Cancer, Oncology

## Abstract

This study aims to evaluate the feasibility and postoperative outcomes of the thoracodorsal artery perforator (TDAP) flap in oncoplastic breast surgery (OBS), incorporating multidimensional assistive techniques. We retrospectively analyzed 14 breast cancer patients treated with TDAP flap OBS from May 2020 to August 2023. Patients were divided into two groups: Group A (first 7 cases) and Group B (last 7 cases). Preoperative perforator localization was performed using color Doppler ultrasound and handheld Doppler, with intraoperative comparisons. Blood perfusion was assessed intraoperatively with indocyanine green (ICG) fluorescence imaging and postoperatively with infrared thermal (IRT) imaging. Patient satisfaction was measured using the BREAST-Q scale. Group A had longer operation times (4.05 ± 0.61 h vs. 3.27 ± 0.31 h, *P* = 0.011) and longer hospitalization (9.14 ± 2.27 days vs. 7.71 ± 1.60 days, *P* = 0.199). No flap necrosis occurred. Preoperative and intraoperative perforator positions were consistent. ICG imaging showed good flap blood supply; edge trimming improved perfusion in two cases. IRT imaging confirmed good blood perfusion in the first 3 days post-op. BREAST-Q scores for psychological health and sexual satisfaction were lower post-op (*P* < 0.001), but no significant differences were found in scores for chest physical health, shoulder and back physical health, or breast satisfaction. While TDAP flap surgery involves a learning curve, it is a highly feasible technique for OBS. Multidimensional assistive technologies significantly enhance preoperative perforator localization and intraoperative flap blood perfusion monitoring, thereby improving flap survival rates and patient satisfaction.

**Supplementary Information:**

The online version contains supplementary material available at 10.1038/s41598-025-95073-z.

## Introduction

Surgical treatment remains a cornerstone in the management of early breast cancer, with a trend towards developing individualized strategies tailored to each patient’s condition. These approaches have expanded the treatment options available to breast cancer patients and have significantly enhanced survival rates^1,2^. As patient expectations for quality of life post-surgery increase, the focus of surgical treatment has shifted from ‘the largest tolerable intervention’ to ‘the smallest effective intervention’. This evolution reflects a move from extensive radical surgeries to modified radical procedures, and more recently, to breast-conserving surgeries (BCS) and breast reconstruction, which have become increasingly popular^3–5^. Nonetheless, both BCS and breast reconstruction require adept oncoplastic surgery techniques to achieve optimal outcomes.

Oncoplastic breast surgery (OBS) aims to enhance postoperative quality of life by addressing breast defects and local chest wall deformities caused by cancer surgery, reshaping the breast curve to provide both physical and psychological benefits^6–8^. This surgery incorporates both volume displacement and volume replacement techniques. The volume displacement technique is often employed in OBS: Type I is used when less than 20% of the breast gland is removed, while Type II is applied for resections between 20% and 50%, including methods such as fish-hook-shaped incision rotating flaps and breast reduction. For cases where over 50% of the breast gland is removed or where Type II techniques lead to significant deformities, volume replacement technology is considered. This method uses local flaps to replace the defect site, achieving breast reconstruction and aesthetic improvement^9^. Among various options, the thoracodorsal artery perforator (TDAP) flap is increasingly utilized in OBS due to its reliable vascular pedicle, ample blood supply, and relatively straightforward procedure^10,11^.

The latissimus dorsi (LD) myocutaneous flap, known for its simplicity and minimal trauma, is widely utilized in OBS due to its effective reconstruction and minimal postoperative complications. Despite its advantages, such as a low impact on disease-free survival rates, the LD myocutaneous flap can potentially lead to back complications and shoulder joint dysfunction^12^. In 1995, the first case of the TDAP flap was introduced as a refinement to address these issues, offering a method to minimize the impact on the LD muscle and improve outcomes^13^. Initially used for soft tissue reconstruction in various anatomical regions, TDAP flaps were later adapted for breast reconstruction by Hamdi et al. in 2004^14^. TDAP flaps provide a significant advantage over traditional LD flaps by preserving muscle function, reducing postoperative complications like shoulder movement restriction and serum swelling, and enhancing cosmetic results.

To further explore the practical application of the TDAP flap in OBS, we conducted a retrospective study on patients who underwent OBS with TDAP flap reconstruction immediately following breast cancer surgery. This study aims to evaluate the clinical effectiveness of TDAP flap surgery by employing various techniques for precise preoperative perforator localization and comprehensive intraoperative assessment of flap blood supply. Our objectives are to assess the clinical effectiveness of TDAP flap surgery, summarize surgical techniques and common postoperative complications, and objectively evaluate patient satisfaction using the BREAST-Q evaluation scale. Through this study, we seek to provide practical guidance for refining TDAP flap techniques in OBS, thereby enhancing the safety and effectiveness of the procedure and ultimately improving patient outcomes and satisfaction in OBS.

## Patients and methods

### Study design and participants

This retrospective study analyzed data from patients diagnosed with invasive breast cancer who underwent OBS with TDAP flap at the Department of Breast Surgery, Quanzhou First Hospital of Fujian Medical University, from May 2020 to August 2023. The inclusion criteria were as follows: (1) Patients with indications for OBS who consented to TDAP flap surgery; (2) Patients requiring local repair due to inability to directly suture or local deformity after tumor resection; (3) Patients with adequate TDAP flap tissue volume for breast tissue defect repair and no prior surgery on the back; and (4) Patients with complete clinicopathological data and willingness to participate in postoperative surveys.The exclusion criteria for patients were as follows: (1) Patients undergoing TDAP flap surgery for non-breast cancer conditions; (2) Patients with serious systemic diseases affecting the heart, brain, liver, kidneys, immune system, or blood system; and (3) Patients with mental illnesses, psychological disorders, or unrealistic expectations not aligned with clinical practice.

Following rigorous screening, 14 patients were included in the study. Based on the chronological order of their surgeries, they were divided into two groups: the first 7 cases were assigned to Group A, and the last 7 cases to Group B.

This study strictly adhered to rigorous medical ethical standards and was granted approval by the Ethics Committee of the First Hospital of Quanzhou, Fujian Medical University (Approval Number: Quan Yi No. 193). The research was conducted in full accordance with the principles outlined in the Declaration of Helsinki (2013 revision). Written informed consent was obtained from all enrolled patients or their authorized family members, emphasizing the paramount importance of ethical considerations in this research endeavor.

### Clinical data collection

Clinical and demographic information of patients was collected, including age, height, BMI, surgical history, comorbidities, chemotherapy history, radiotherapy history, smoking history, tumor size, pathological type, and stage. Surgical-related data included the duration of surgery, length of hospitalization, intraoperative bleeding volume, flap size, and incision pain score. Postoperative complications were also documented, including donor site serum swelling, wound infection, wound dehiscence, fat liquefaction, flap necrosis, and shoulder joint functional injury. Defect volume calculation: Tumor resection volume was estimated using the formula for an ellipsoid, V = (4/3)πabc, where a, b, and c are the radii of the resected specimen. Flap design: The flap dimensions (length, width, and thickness) were designed to exceed the defect volume by 10–15% to account for tissue contraction. Intraoperative adjustments were made based on ICG perfusion feedback.

The duration of surgery is defined as the total time from the initiation of anesthesia to the completion of skin suturing. The length of hospitalization is measured from the day of surgery to the day of discharge. Incision pain scores were evaluated using the Numerical Rating Scale (NRS)^15^, which assesses pain on postoperative days 1, 2, and 3. Pain levels are rated on a scale of 0 to 10, with 0–3 indicating mild pain, 4–7 indicating moderate pain, and 8–10 indicating severe pain. Two weeks post-surgery, shoulder joint function was evaluated by measuring arm flexion (forward tilt) and abduction (lateral elevation). A reduction of 25° or more in shoulder joint mobility is considered indicative of impaired shoulder function^16^. Serum swelling in the donor site, occurring within 24 h to 1 month post-surgery, was confirmed through drainage volume, puncture and aspiration, or ultrasound results.

### Localization of TDAP flap perforator before surgery

**Fig. 1:**
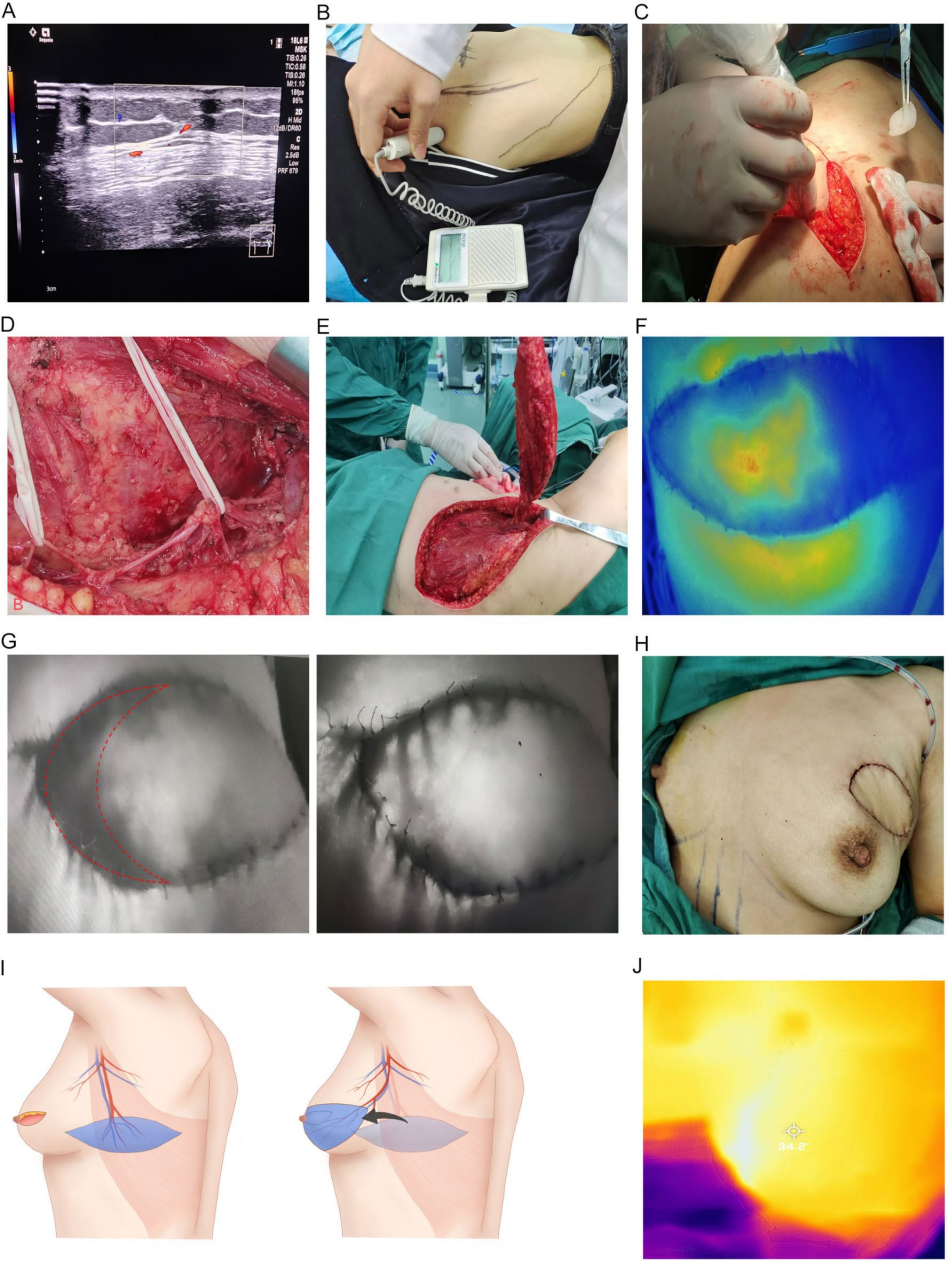
The whole process of OBS using TDAP, including preoperative Doppler perforator positioning (**A-C**), intraoperative perforator condition (**D-E**), intraoperative ICG detection (**F-G**), operation completion (**H**), flap transfer schematic diagram (**I**) and postoperative IRT flap monitoring (**J**).

Color duplex ultrasound (CDUS) is used to evaluate the blood supply of the TDAP flap, displaying colored blood flow signals on two-dimensional ultrasound images (Fig. 1A). The Philips Aff50-09 instrument with a linear high-frequency probe (18L6) operating at a frequency of 10–18 MHz was employed in this study. CDUS helps determine the exit point, inner diameter, and direction of the TDAP perforators, which are then marked on the body surface. This preoperative localization of the TDAP flap was carried out jointly by an ultrasound specialist and a breast surgeon.

Additionally, handheld Doppler HD was utilized to locate the perforating vessels of the TDAP flap (Fig. 1B). By correlating the surface projection of the anatomical location of the blood vessels with the positioning points marked using color Doppler, the perforators were detected progressively from the head side to the foot side along the main trunk of the thoracodorsal artery. These were tracked, marked, and compared with the perforators identified by color Doppler to select and mark the optimal perforating branch on the body surface.

## Operation procedure

### Design of TDAP flap

The design of the flaps primarily depends on the location of potential perforators, the size of the defects, and the ability to close the donor site. During flap design, the patient should be seated with both arms at their sides and hands on their waist. The patient is then asked to actively contract their back muscles while a marker is used to outline the leading edge of the contracted LD muscle. The anatomical location where the descending branch of the thoracodorsal artery passes through the muscle is marked on this outline, typically about 8 cm below the posterior axillary wall and 2–3 cm medial to the lateral edge of the LD muscle^17^. The pivot point is determined preoperatively as the intersection of the thoracodorsal artery’s descending branch and the lateral edge of the latissimus dorsi (LD) muscle, approximately 8 cm below the posterior axillary fold. A handheld Doppler is used to identify and mark 2–3 suitable perforating branches within this area. Intraoperatively, this is confirmed using handheld Doppler and correlated with preoperative color Doppler markings. The final flap size is determined based on the dimensions of the breast defect, while the width of the flap is designed to ensure the donor site can be closed directly. Depending on the defect location (e.g., upper vs. lower quadrants), the flap is designed with a slight caudal or cranial bias to account for pendulum rotation. For example, defects in the upper quadrant require the flap’s pivot point to be positioned slightly lower on the back to ensure tension-free rotation.

### OBS with TDAP flap

Preoperative Doppler imaging was used to position the TDAP flap, and a transverse shuttle-shaped flap was designed based on this landmark. An incision was made along the preoperative landmark line, through the skin and subcutaneous fat to the LD muscle. The tissue flap was then elevated from the inner axillary line along the surface of the LD muscle. The anterior edge of the LD muscle was located, and handheld Doppler ultrasound was used during surgery to identify the descending branch of the thoracodorsal artery (Fig. 1C). This was confirmed with the preoperative ultrasound positioning point to accurately locate the pivot point.

The site where the thoracodorsal artery branches out of the LD muscle was carefully investigated. The separation continued along the branch towards the thoracodorsal artery pedicle (Fig. 1D), with careful attention to protecting the thoracic and dorsal nerves. The pedicle of the thoracodorsal artery was fully freed (Fig. 1E), resulting in a complete pedicled TDAP flap.

During the operation, Indocyanine Green Fluorescence Angiography (ICG-FA) was utilized to assess the real-time blood supply of the TDAP flaps (Fig. 1F). Areas of the flap with poor blood supply were trimmed and re-evaluated with ICG-FA (Fig. 1G). A subcutaneous tunnel was created near the armpit and original surgical site, through which the flap was transferred to the defect area on the chest. The flap was sutured and fixed to the surrounding glandular tissue, and the incision was closed (Fig. 1H). A schematic diagram of the TDAP flap transfer is shown in Fig. 1I. On the 1st, 2nd and 3rd day after operation, the blood supply of the flap was monitored by infrared thermography (IRT). Make the infrared thermal imager perpendicular to the flap in the operation area, about 20 cm above the flap, and dynamically observe the changes of the thermal image of the flap and the surrounding area (Fig. 1J).

### Patient satisfaction assessment

This study utilized the BREAST-Q Recon V2.0 Chinese CN (L) Breast Reconstruction Module as the Patient Reported Outcome Measure (PROM) tool. BREAST-Q has significantly enhanced research on breast surgery satisfaction from the patient’s perspective^18,19^. The study focused on five dimensions from the postoperative scale: mental well-being, sexual well-being, chest and back physical health, shoulder and back physical health, and breast satisfaction.

### Statistical analysis

The clinical data collected from patients was analyzed using SPSS 26.0 and GraphPad Prism 8.0 software. Measurement data are expressed as mean ± standard deviation ($\:\stackrel{-}{x}$± s). For metric data that conform to a normal distribution, the t-test will be employed. A P-value of < 0.05 will be considered statistically significant.

## Results

### General information of patients

The age range of patients in this study was 28 to 70 years, with a median age of 50 years. Group A had a median age of 46 years, while Group B had a median age of 50 years. The patients’ BMI ranged from 20 to 28, with a median BMI of 24. Group A had a median BMI of 22, and Group B had a median BMI of 24. There were two cases with a history of prior surgery (one in each group), two cases with a history of diabetes (one in each group), three cases with hypertension (one in Group A and two in Group B), and five cases with a history of neoadjuvant chemotherapy (three in Group A and two in Group B). All patients had no history of radiation therapy or smoking (Table S1).

Among the patients, there were four cases of T1 tumors (some with peripheral malignant calcifications), including three in Group A and one in Group B; nine cases of T2 tumors (four in Group A and five in Group B); and one case of T3 tumor (Group B). Thirteen cases had a tumor-to-breast volume ratio of 20–50% (seven in Group A and six in Group B), and one case (Group B) had a ratio exceeding 50%. Pathologically, 12 patients were diagnosed with non-specific invasive breast cancer (six cases in Group A and six cases in Group B), one case with high-grade ductal carcinoma in situ (Group A), and one case with mucinous carcinoma (Group B). Postoperative pathological staging included six cases in Stage IIA (four in Group A and two in Group B), three cases in Stage IIB (one in Group A and two in Group B), four cases in Stage IIIA (two in Group A and two in Group B), and one case in Stage IIIB (Group B) (Table 1).

**Table 1:** General information of patients.

	Items	Whole population, *n*(%)	Group A(%)	Group B(%)
Tumo size	T1(≤ 2 cm)	4 (28.57)	3 (42.86)	1 (14.28)
	T2(> 2 cm, ≤ 5 cm)	9 (64.29)	4 (57.14)	5 (71.42)
	T3(> 5 cm)	1 (7.14)	0 (0)	1 (14.28)
Ratio of tumor to breast	20–50%	13 (92.86)	7 (100.00)	6 (85.72)
	> 50%	1 (7.14)	0 (0)	1 (4.28)
Pathology	non-specific invasive breast cancer	12 (85.72)	6 (85.72)	6 (85.72)
	high-grade ductal carcinoma in situ	1 (7.14)	1 (14.28)	0 (0)
	mucinous carcinoma	1 (7.14)	0 (0)	1 (14.28)
Stage	IIA	6 (42.86)	4 (57.14)	2 (28.57)
	IIB	3 (21.43)	1 (14.29)	2 (28.57)
	IIIA	4 (28.57)	2 (28.57)	2 (28.57)
	IIIB	1 (7.14)	0 (0)	1 (14.29)

### Relevant surgical and flap parameters

The anatomical positions of the perforating vessels identified preoperatively in all 14 patients were consistent with those observed during surgery, with no anatomical variations noted. Intraoperative ICG-FA results indicated that all patients had adequate flap blood supply, although two patients initially exhibited poor blood supply at the flap edges. Following trimming of the flap edges, ICG imaging confirmed improved blood supply. IRT imaging showed that the flap blood perfusion was good in all patients in the first 3 days after operation.

**Fig. 2:**
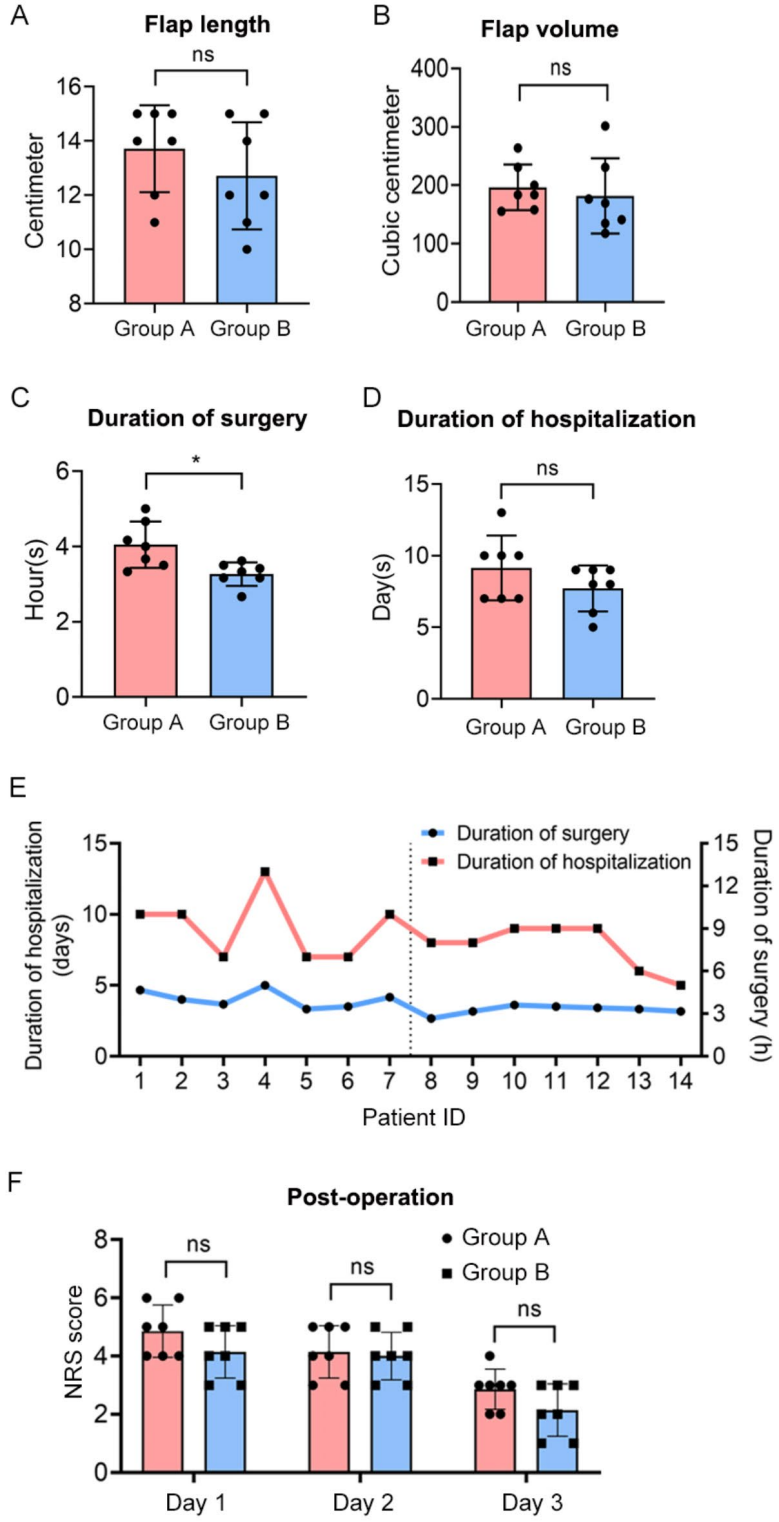
Comparison of relevant surgical and flap parameters. Comparison of flap length, flap volume, duration of surgery and duration of hospitalization in Group A and Group B, respectively (**A-D**). The details of duration of surgery and hospitalization time for each patient (**E**). The comparison of postoperative NRS score for Group A and Group B from day 1 to day 3 (**F**).

The average flap length across all patients was 13.21 ± 1.80 cm. The average flap lengths for Group A and Group B were 13.71 ± 1.60 cm and 12.71 ± 1.98 cm, respectively, with no statistically significant difference between the two groups. The average flap volume for all patients was 189.13 ± 51.74 cm³, with Group A averaging 196.52 ± 39.14 cm³ and Group B 181.74 ± 64.36 cm³. There was no statistically significant difference in flap volumes between the two groups (Fig. 2A and B and Table S2).

The average duration of surgery for all patients was 3.69 ± 0.63 h. Group A had a significantly longer average surgery duration of 4.05 ± 0.61 h compared to Group B, which averaged 3.27 ± 0.31 h (*P* = 0.011). The average hospitalization duration for all patients was 8.43 ± 2.03 days. Group A had a longer average hospitalization of 9.14 ± 2.27 days compared to Group B’s 7.71 ± 1.60 days, but this difference was not statistically significant (*P* = 0.199) (Fig. 2C and D and Table S2). The duration of surgery and hospitalization time for each patient is detailed in Fig. 2E.

On the 1st, 2nd, and 3rd days post-surgery, the incision pain scores in Group A were slightly higher than in Group B, though the difference was not statistically significant (Fig. 2).

### Postoperative complications

Among the 14 patients, there were no cases of hematoma, wound dehiscence, or seroma at the donor site, and none of the patients experienced flap necrosis. No cases of shoulder joint dysfunction were observed in any patient. However, there was one case of wound infection and one case of fat liquefaction, both occurring in Group A (Table S3 and Figure S1). These complications were effectively managed and resolved with intensified dressing changes and oral antibiotics.

### Patient reported outcome

**Fig. 3:**
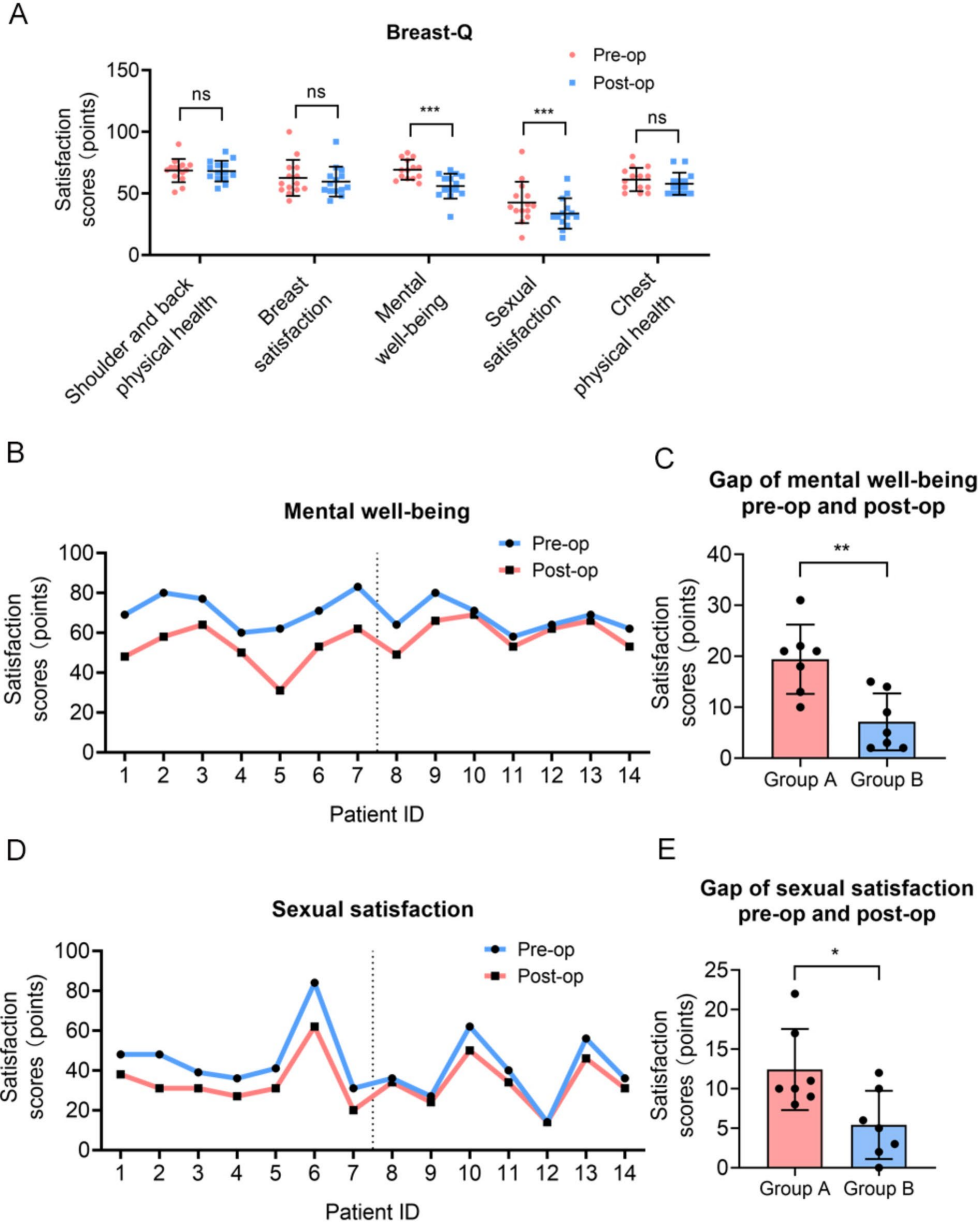
Comparison of the BREAST-Q score in preopration and postoperation. The BREAST-Q score for shoulder and back physical health, breast satisfaction, mental well-being, sexual satisfaction and chest physical health (**A**). The satisfaction scores of mental well-being for each patient before and after surgery, and gap for patients in Group A and Group B (**B-C**). The satisfaction scores of sexual satisfaction for each patient before and after surgery, and gap for patients in Group A and Group B (**D-E**).

All patients in this study were followed up for 6 to 18 months, with a median follow-up time of 12 months. The BREAST-Q score for mental well-being postoperatively was 56.00 ± 10.04, significantly lower than the preoperative score of 69.29 ± 8.13, with a statistically significant difference (*P* < 0.001, Fig. 3A). Figure 3B illustrates the satisfaction scores of each patient before and after surgery, showing that the mental well-being gap for patients in Group A was greater than that in Group B, with a statistically significant difference (*P* < 0.01, Fig. 3C). The BREAST-Q score for postoperative sexual satisfaction was 33.78 ± 12.34, markedly lower than the preoperative score of 42.71 ± 16.82, with a statistically significant difference (*P* < 0.001). Figure 3D displays the satisfaction scores of each patient before and after surgery, indicating that the sexual well-being gap for patients in Group A was greater than in Group B, with a statistically significant difference (*P* < 0.05, Fig. 3E). There were no statistically significant differences in postoperative shoulder and back physical health, breast satisfaction, and chest physical health compared to preoperative levels (Fig. 3A, Table S4).

### Typical case

**Fig. 4:**
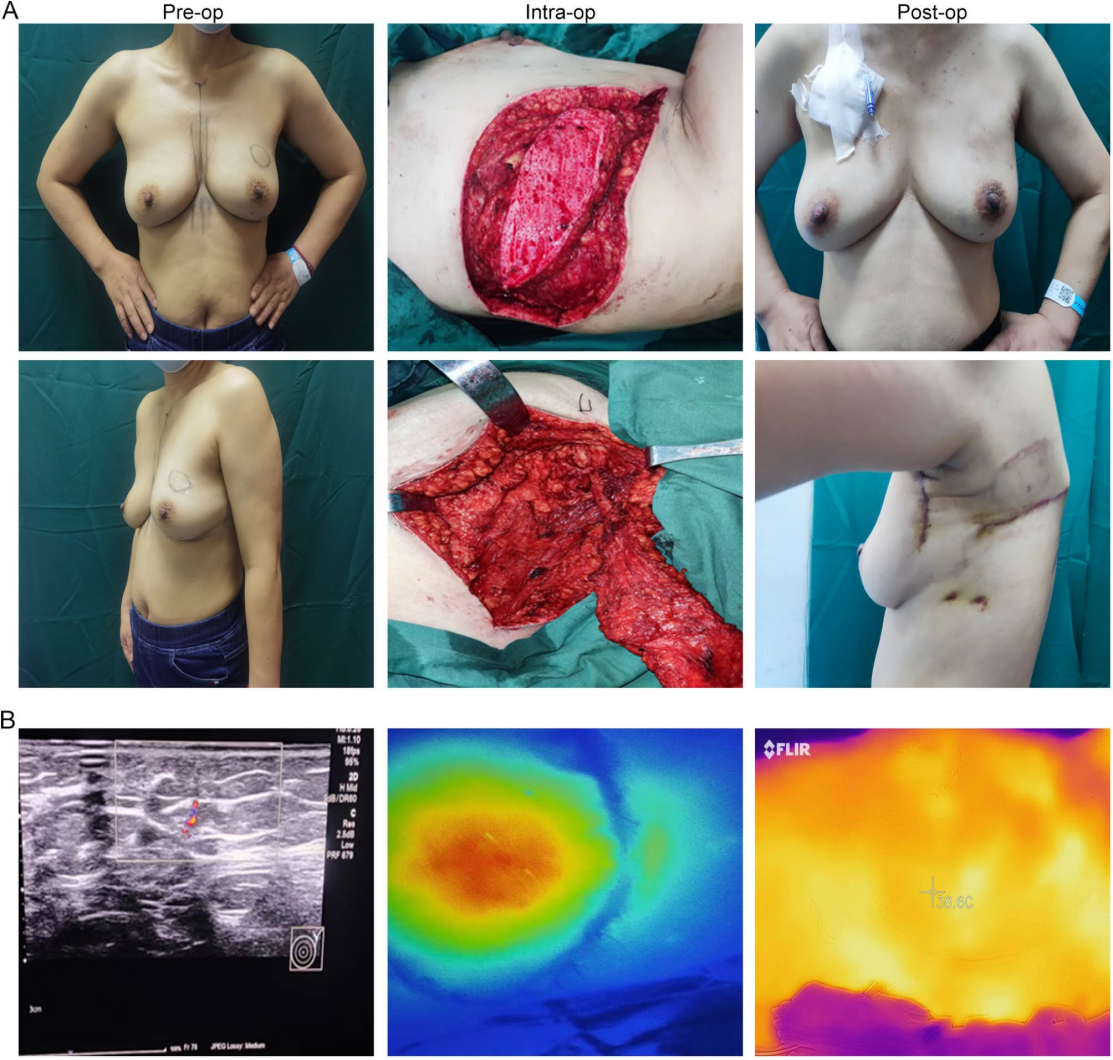
Relevant images of patients accept OBS with TDAP flap (fully embedded) before, during and after operation. Imaging before, during, and one month after the surgery (**A**), and preoperative perforator positioning, intraoperative ICG, and postoperative IRT (**B**) detection.

Case 1: A 50-year-old female patient presented with a left breast tumor that had been identified for over one month. She underwent OBS with a TDAP flap, fully embedded. The postoperative pathology confirmed non-specific invasive breast cancer (pT2N0M0, Stage IIA). Imaging before, during, and one month after the surgery is shown in Fig. 4A. Preoperative color Doppler and handheld Doppler were used to locate the perforating branches, and intraoperatively ICG-FA and postoperatively IRT imaging demonstrated adequate blood supply to the flap (Fig. 4B).

**Fig. 5:**
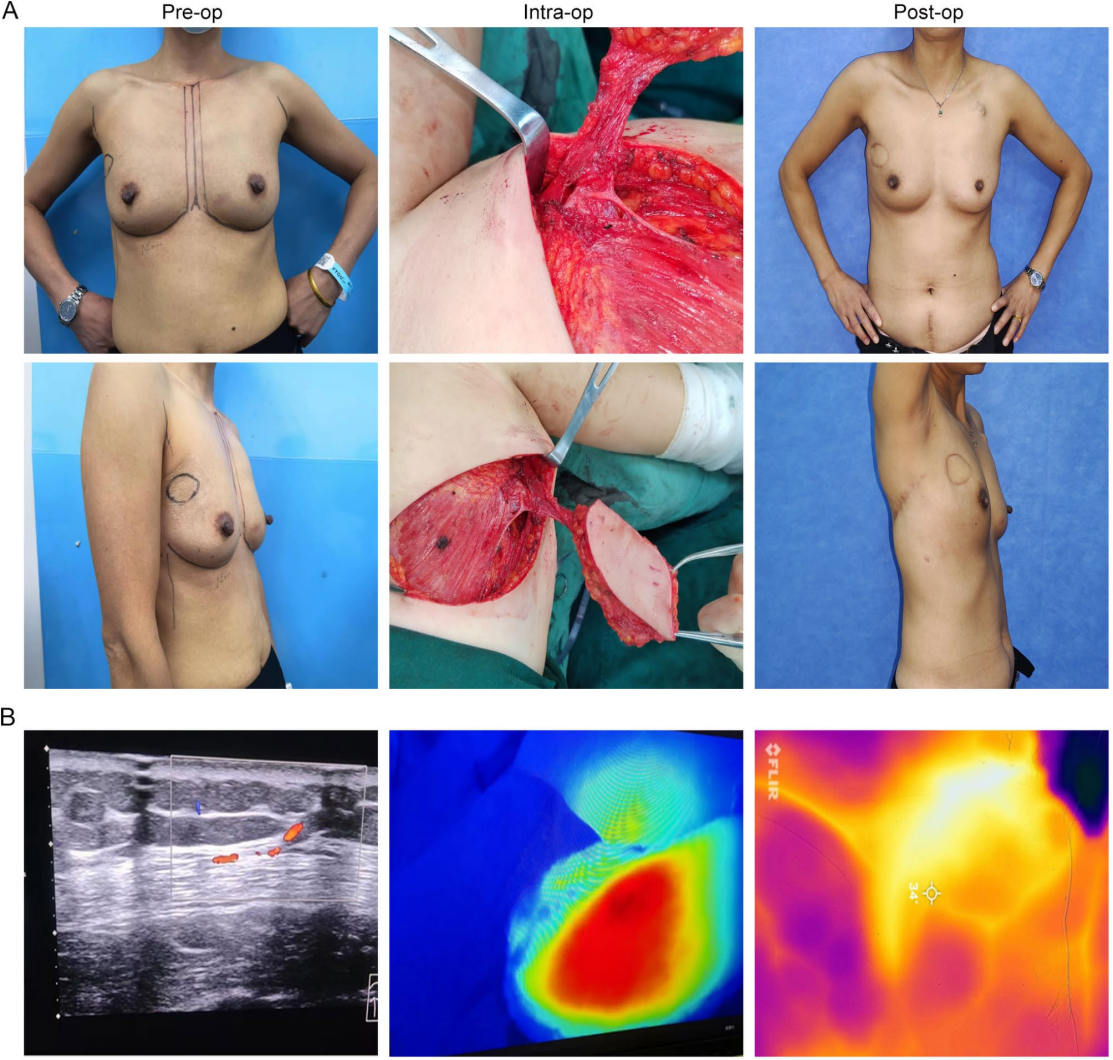
Relevant images of patients accept OBS with TDAP flap (preserving the observation window) before, during and after operation. Imaging before, during, and one year after the surgery (**A**), and preoperative perforator positioning, intraoperative ICG, and postoperative IRT (**B**) detection.

Case 2: A 44-year-old female patient presented with a right breast mass that had been present for over 4 years. She underwent OBS with a TDAP flap, preserving the skin island. Preoperative, intraoperative, and 1-year postoperative imaging are illustrated in Fig. 5A. Preoperative color Doppler and handheld Doppler were utilized to identify the perforator’s location, and intraoperative ICG-FA and postoperatively IRT imaging demonstrated a robust blood supply to the TDAP flap (Fig. 5B).

## Discussion

Currently, BCS combined with postoperative radiotherapy is the standard treatment for early-stage breast cancer. Numerous studies have demonstrated that BCS offers overall survival and disease-free survival rates comparable to those of mastectomy in early breast cancer patients. The optimal oncological outcome of BCS hinges on the complete excision of malignant tumors and achieving negative surgical margins. Building on this foundation, OBS addresses local defects following tumor resection, mitigates the risk of breast deformities, and enhances bilateral breast symmetry, thereby achieving both reconstructive and aesthetic outcomes.

The TDAP flap is primarily utilized for partial soft tissue reconstruction following BCS. There are limited reports on the application of TDAP flaps post-mastectomy. Angrigiani’s “extended” harvest technique of TDAP flaps, akin to LD flaps, achieves the desired breast reconstruction volume without the need for implants^20^. The primary drawback of this procedure is the relatively long donor site scar, which extends significantly beyond the bra’s coverage area. Considering postoperative complications and superior cosmetic outcomes^11,21^, the TDAP flap offers advantages over traditional LD flaps, as it preserves the LD muscle’s function more effectively. However, it is noteworthy that there was one case of wound infection and one case of fat necrosis in this study, both occurring in Group A (early stage of surgical experience accumulation), likely related to insufficient surgical experience. Both complications were effectively managed and resolved satisfactorily. With increasing surgical experience, there will be greater proficiency in preventing and managing complications.

The anatomical position of the TDAP flap is relatively constant, with the flap primarily supplied by the proximal perforator of the descending branch of the thoracodorsal artery^13,22,23^. However, anatomical variations between individuals can occur, such as multiple small perforating branches or a dominant perforating branch originating from a vessel other than the descending branch of the thoracodorsal artery^24–26^. Unlike conventional flaps, perforator flap surgery necessitates the dissection of small perforator vessels^27,28^, making preoperative vessel identification and labeling crucial to reduce complications. It is essential to determine an advantageous perforating branch before surgery. Currently, color Doppler ultrasound and handheld Doppler are the most widely used preoperative techniques for locating perforating vessels^29,30^, being non-invasive and safe. Studies have shown that the accuracy of preoperative Doppler examination reaches 90%^30^. This study utilized color Doppler combined with handheld Doppler to locate perforating vessels preoperatively, enabling an understanding of the thoracic and dorsal vascular anatomy for each patient, providing an anatomical basis for flap design, improving surgical accuracy, and reducing surgical complications. During the operation, patients with unclear perforator positioning can undergo additional localization to accurately identify the perforator, avoid damage, and reduce the incidence of postoperative flap ischemic necrosis.

ICG-FA technology enables surgeons to observe flap blood flow in real time, accurately assess the blood perfusion^31^, and guide surgical procedures. IRT imaging technology monitors temperature changes in the flap, indirectly reflecting its postoperative blood perfusion^32–34^. Utilizing both ICG-FA and IRT imaging, surgeons can rapidly evaluate the blood perfusion during and after surgery, promptly identify and address potential issues of inadequate perfusion, and thereby improve flap survival rates. This study employed ICG-FA and IRT technology, in addition to traditional clinical judgment and flap examination (including color, capillary refill, and skin bleeding), to determine perfusion status. This approach allowed for real-time evaluation of blood perfusion in TDAP flaps, significantly reducing the risk of postoperative flap necrosis.

The BREAST-Q score for postoperative mental health was significantly higher than that before surgery, with *P* < 0.05 (Fig. 3A and B), indicating a statistically significant improvement. This may be related to factors such as physical changes during surgery, changes in breast appearance, postoperative psychological adaptation, and postoperative incision pain. The postoperative breast satisfaction score was slightly lower than the preoperative score, but with *P* > 0.05 (Fig. 3A), the difference was not statistically significant. The slight decrease in postoperative breast satisfaction score may be due to discrepancies between patients’ expectations and actual outcomes regarding breast appearance, higher preoperative expectations, and individual differences in the surgical process. Research reports indicate that preoperative depression, anxiety levels, and psychological vulnerability to abnormal pain perception are significantly correlated with greater postoperative pain intensity, which may reduce satisfaction with physical health^35^.

In this study, the BREAST-Q scores for shoulder, back, and chest health were lower postoperatively compared to preoperatively, but the differences were not statistically significant (*P* > 0.05). Additionally, there was no significant change in shoulder scores post-surgery, likely because TDAP flap surgery does not impair shoulder joint function, as observed in the 14 patients studied. Overall, the BREAST-Q scores for postoperative chest physical health, shoulder and back physical health, and breast satisfaction showed no significant difference from preoperative levels, indicating that the TDAP flap has achieved satisfactory outcomes in postoperative quality of life and patient satisfaction in breast tumor plastic surgery. Furthermore, the duration of surgery for patients in Group A (4.05 ± 0.61 h) was significantly longer than for Group B patients (3.27 ± 0.31 h), with a statistically significant difference (*P* < 0.05), suggesting a learning curve associated with TDAP flap surgery. As surgical experience and skills improve, it is expected that the surgical time will decrease. In details, improved familiarity with the TDAP anatomy reduced time spent dissecting the vascular pedicle (average reduction: 25 min), greater reliance on preoperative color Doppler (vs. intraoperative exploration) streamlined perforator identification, and transition from interrupted to continuous suturing for donor-site closure saved ~ 15 min per case. Although the duration of hospitalization for Group A (9.14 ± 2.27 days) was slightly longer than for Group B (7.71 ± 1.60 days), the difference was not statistically significant (*P* = 0.199). This similarity in hospitalization time may be attributed to the need for close postoperative observation to ensure proper flap recovery. However, a trend towards shorter hospitalization times was noted in the later patients (Fig. 2E), and with further experience, it is anticipated that hospitalization duration can be reduced, which will positively impact patient treatment experiences and reduce medical costs.

Although TDAP flaps have been previously described in OBS, our study uniquely integrates multidimensional assistive technologies (color Doppler, handheld Doppler, ICG fluorescence angiography, and infrared thermal imaging) to optimize preoperative perforator localization, intraoperative blood perfusion monitoring, and postoperative flap assessment. While prior studies have utilized individual technologies (e.g., ICG or Doppler), our systematic combination of these tools ensures higher precision in perforator selection, reduces intraoperative decision-making time, and minimizes postoperative complications.

This study has several limitations. First, the small sample size (*n* = 14) may limit the generalizability of our findings. However, our cohort size aligns with prior TDAP flap studies (e.g., Amin et al., 2017^11^: (*n* = 12); Gatto et al., 2022^21^: (*n* = 18), and the consistent outcomes across groups support the validity of our conclusions. Second, the single-center, retrospective design introduces potential selection bias. Future multicenter prospective studies with larger cohorts are warranted to validate our results. Lastly, long-term follow-up data on aesthetic outcomes and recurrence rates are needed to fully assess the TDAP flap’s oncological safety.

Overall, the TDAP flap is a highly feasible surgical technique for breast tumor plastic surgery. Various auxiliary technologies play a crucial role in preoperative precise positioning and intraoperative monitoring of flap blood supply, thereby enhancing the survival rate of TDAP flaps and leading to improved postoperative breast satisfaction and quality of life for patients. Despite a certain learning curve associated with TDAP flap surgery, as the number of procedures increases, both surgical time and hospital stay tend to decrease, and experience in preventing and managing complications improves. This progression will contribute to the broader application and optimization of this surgical technique for patients.

## Electronic supplementary material

Below is the link to the electronic supplementary material.

Supplementary Material 1

## Data Availability

The data that support the findings of this study are available from the corresponding author upon reasonable request.
