# Supplementary material for: Optimizing thoracodorsal artery perforator flap outcomes in oncoplastic breast surgery: multidimensional assistive techniques mitigate learning curve and enhance feasibility
Source: Sci Rep. 2025 Mar 29;15:10937. doi: 10.1038/s41598-025-95073-z (PMC11954998; doi:10.1038/s41598-025-95073-z)
Supplement: Supplementary file 1 — Supplementary Material 1 [file 41598_2025_95073_MOESM1_ESM.docx]

**Supplementary tables and figure**

**Table S1.** General patient information

| Characteristics | Category | Whole population, n (%) | Group A (%) | Group B (%) |
| --- | --- | --- | --- | --- |
| Age | average | 50.86 | 47.71 | 54 |
|  | median | 50 | 46 | 50 |
| BMI | average | 23.86 | 23.14 | 24.57 |
|  | median | 24 | 22 | 24 |
| History of surgery | Yes | 2（14.28） | 1（7.14） | 1（7.14） |
|  | No | 12（85.72） | 6（42.86） | 6（42.86） |
| History of diabetes | Yes | 2（14.28） | 1（7.14） | 1（7.14） |
|  | No | 12（85.72） | 6（42.86） | 6（42.86） |
| History of hypertension | Yes | 3（21.43） | 1（7.14） | 2（14.28） |
|  | No | 11（78.57） | 6（42.86） | 5（35.72） |
| History of radiation therapy | Yes | 0（0） | 0（0） | 0（0） |
|  | No | 14（100） | 7（50） | 7（50） |
| History of chemotherapy | Yes | 5（35.72） | 3（21.43） | 2（14.28） |
|  | No | 9（64.28） | 4（28.57） | 5（35.72） |
| Smoking history | Yes | 0（0） | 0（0） | 0（0） |
|  | No | 14（100） | 7（50） | 7（50） |

**Table S2.** Comparison of flap length, flap volume, duration of surgery and duration of hospital stay between the two groups of patients

| Items | Whole population | Group A | Group B | *P* |
| --- | --- | --- | --- | --- |
| Flap length (cm) | 13.21±1.80 | 13.71±1.60 | 12.71±1.98 | 0.319 |
| Flap volume (cm^3^) | 189.13±51.74 | 196.52±39.14 | 181.74±64.36 | 0.613 |
| Duration of surgery (h) | 3.69±0.63 | 4.05±0.61 | 3.27±0.31 | 0.011 |
| duration of hospitalization (d) | 8.43±2.03 | 9.14±2.27 | 7.71±1.60 | 0.199 |

**Table S3.** Postoperative complications

| Category | number | Ratio (%) |
| --- | --- | --- |
| Hemorrhage | 0 | 0 |
| Seroma at donor site | 0 | 0 |
| Wound infection | 1 | 7.14 |
| Wound cleft | 0 | 0 |
| Fat liquefaction | 1 | 7.14 |
| Complete flap necrosis | 0 | 0 |
| Partial flap necrosis | 0 | 0 |
| Shoulder functional impairment | 0 | 0 |

**Table S4.** The patient-reported outcomes of breast reconstruction using the BREAST-Q .

| Items | Pre-operation | Post-operation | *P* |
| --- | --- | --- | --- |
| Mental well-being | 69.29±8.13 | 56.00±10.04 | ≤ 0.001 |
| Sexual satisfaction | 42.71±16.82 | 33.78±12.34 | ≤ 0.001 |
| Chest physical health | 61.36±9.40 | 57.93±8.96 | 0.167 |
| shoulder and back physical health | 68.64±9.44 | 68.14±8.39 | 0.569 |
| Breast satisfaction | 62.64±14.63 | 59.71±12.13 | 0.073 |


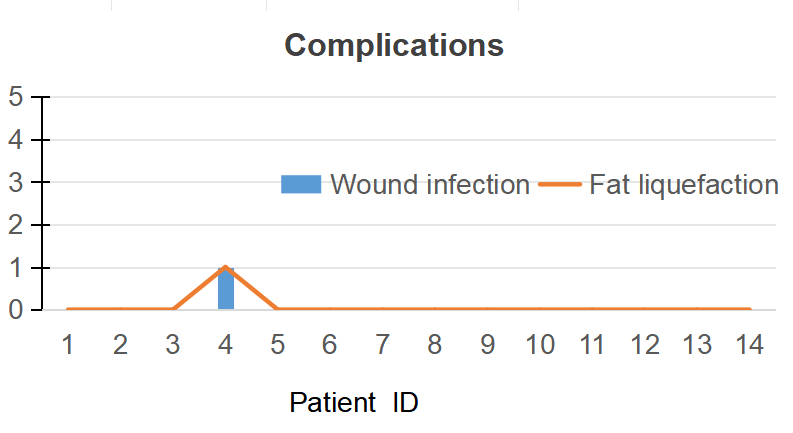


**Figure S1**. The incidence of complications in each patient.
